# Novel K_2_Ti_8_O_17_ Anode via Na^+^/Al^3+^ Co-Intercalation Mechanism for Rechargeable Aqueous Al-Ion Battery with Superior Rate Capability

**DOI:** 10.3390/nano11092332

**Published:** 2021-09-08

**Authors:** Qiangqiang Feng, Yanyan Liu, Jitong Yan, Wei Feng, Shaozheng Ji, Yongfu Tang

**Affiliations:** 1 Hebei Key Laboratory of Applied Chemistry, College of Environmental and Chemical Engineering, Yanshan University, Qinhuangdao 066004, China; f2394713046@163.com (Q.F.); yanjitong@outlook.com (J.Y.); fw980240907@163.com (W.F.); 2 Materials and Nano Physics, School of Engineering Sciences, KTH Royal Institute of Technology, SE-100 44 Stockholm, Sweden; jshaoz@kth.se; 3 School of Physics, Ultrafast Electron Microscopy Laboratory, Nankai University, Tianjin 300071, China

**Keywords:** aluminum ion battery, K_2_Ti_8_O_17_ anode, discharge–charge mechanism

## Abstract

A promising aqueous aluminum ion battery (AIB) was assembled using a novel layered K_2_Ti_8_O_17_ anode against an activated carbon coated on a Ti mesh cathode in an AlCl_3_-based aqueous electrolyte. The intercalation/deintercalation mechanism endowed the layered K_2_Ti_8_O_17_ as a promising anode for rechargeable aqueous AIBs. NaAc was introduced into the AlCl_3_ aqueous electrolyte to enhance the cycling stability of the assembled aqueous AIB. The as-designed AIB displayed a high discharge voltage near 1.6 V, and a discharge capacity of up to 189.6 mAh g^−1^. The assembled AIB lit up a commercial light-emitting diode (LED) lasting more than one hour. Inductively coupled plasma–optical emission spectroscopy (ICP-OES), high-resolution transmission electron microscopy (HRTEM), and X-ray absorption near-edge spectroscopy (XANES) were employed to investigate the intercalation/deintercalation mechanism of Na^+^/Al^3+^ ions in the aqueous AIB. The results indicated that the layered structure facilitated the intercalation/deintercalation of Na^+^/Al^3+^ ions, thus providing a high-rate performance of the K_2_Ti_8_O_17_ anode. The diffusion-controlled electrochemical characteristics and the reduction of Ti^4+^ species during the discharge process illustrated the intercalation/deintercalation mechanism of the K_2_Ti_8_O_17_ anode. This study provides not only insight into the charge–discharge mechanism of the K_2_Ti_8_O_17_ anode but also a novel strategy to design rechargeable aqueous AIBs.

## 1. Introduction

Rechargeable lithium-ion batteries as promising energy storage devices have been widely applied in portable electronic devices and electric vehicles [1,2]. However, lithium-ion batteries may be not the best choice for large-scale energy storage applications and practical power grids, concerning its uneven distribution and the long-term unavailability of lithium resources. Consequently, ‘beyond-Li-ion’ batteries, including Mg-based batteries [3], Zn-based batteries [4,5], Na-based batteries [6], and Al-based batteries [7,8,9], have emerged as promising alternatives for electrochemical energy storage. Therein, aluminum-based batteries (ABs) exhibit considerable advantages in terms of cost-effectiveness, safety, and high theoretical specific capacity due to the abundant aluminum reserves, nonflammability, light weight, and three-electron redox properties [10,11,12]. The trivalent aluminum ion, in principle, provides three reactive electrons involved in the electrochemical processes ($Al^{3+}+3e^{-}↔Al$), endowing a high volumetric capacity of 8040 mAh cm^−3^ and a gravimetric capacity of 2980 mAh g^−1^ [13]. Furthermore, aluminum metal can be handled in ambient atmosphere, thus facilitating cell fabrication and resulting in a high level of safety of electrochemical storage systems [14].

According to the electrolytes, ABs are classified into aqueous and nonaqueous systems. In nonaqueous ABs, the aluminum metal can be employed directly as the anode [12,15]. However, high-cost ionic liquid electrolytes or complicated molten salt electrolytes limit the wide practical application of nonaqueous ABs [16,17]. Therefore, a growing number of researchers have turned their attention to the aqueous ABs due to the reduced cost and convenient operation of aqueous electrolytes. Unfortunately, the reversible charging process of ABs in aqueous electrolytes is hindered by the competitive hydrogen evolution reaction (HER). The lower redox potential of Al^3+^/Al compared to that of H^+^/H_2_ implies that the Al (aluminum) metal anode can only be used in primary ABs rather than rechargeable ABs for aqueous electrolytes. Due to thermodynamics, the development of appropriate intercalation/deintercalation-type anode materials plays a critical role in the assembly of rechargeable aqueous ABs [17,18,19], which can be called aluminum-ion batteries (AIBs). Liu et al. proposed an aqueous AIB prototype using a TiO_2_ anode and AlCl_3_ electrolyte for the first time, evidencing the reversible intercalation/deintercalation of Al^3+^ ions into/from TiO_2_ [15]. Subsequently, they used another AIB device using TiO_2_ as the anode, while copper hexacyanoferrate and Al_2_(SO_4_)_3_ were employed as the cathode and electrolyte, respectively. It delivered an acceptable discharge capacity of 21 mAh g^−1^ with a discharge voltage of 1.6 V [20]. Thus, TiO_2_ is considered a promising candidate as an aqueous AIB anode. An enhanced rate capability or better cycling performance was required via selecting a superior electrolyte or adding H_2_ evolution inhibitors for the AIB system [21]. 

Herein, we developed a novel aqueous AIB system consisting of a layered-structure K_2_Ti_8_O_17_ anode obtained via a facile hydrothermal method. An AlCl_3_ aqueous solution with NaAc additive was employed as the electrolyte, and activated carbon (AC) was used as the cathode. The aqueous AIB delivered an acceptable discharge capacity of 189.6 mAh g^−1^ based on the anode materials. An enhanced rate performance and high cycling stability were achieved for the assembled AIB. Inductively coupled plasma–optical emission spectroscopy (ICP-OES), high-resolution transmission electron microscopy (HRTEM), and K-edge X-ray absorption near-edge spectroscopy (XANES) techniques were conducted to provide evidence of the mechanism descriptions of Na^+^/Al^3+^ ion intercalation/deintercalation in aqueous AIB, inspiring a great potential to explore promising aqueous AIB anode materials. 

## 2. Materials and Methods

### 2.1. Chemical Reagents

Titanium foil with a purity of 99% was purchased from Qingyuan Metal Materials Co., Ltd. (Xingtai, China) Potassium hydroxide (KOH), hydrochloric acid (HCl), hydrofluoric acid (HF), and concentrated nitric acid (HNO_3_) were all purchased from Tianjin FengChuan Chemical Reagent Technology Corp. Ltd. (Tianjin, China) Aluminum chloride hexahydrate (AlCl_3_·6H_2_O) was purchased from Qinhuangdao Reagent Plant (Shanghai, China). Sodium acetate (NaAc) and activated carbon (AC) were purchased from Aladdin Corp., Ltd. (Shanghai, China) All these reagents were analytically pure (AR) and used without further purification. 

### 2.2. Preparation of K_2_Ti_8_O_17_ Electrode

The binder-free K_2_Ti_8_O_17_ electrode material was prepared via a two-step method, including a hydrothermal reaction combined with a post-pyrolysis process. First, titanium foil with a thickness of 0.1 mm was cut into a patch with an area of 2.0 × 0.5 cm^2^. Then, ultrasonic treatments were sequentially carried out in acetone, ethanol, and deionized water for 20 min. The clean titanium foil was immersed in a mixture of hydrofluoric acid, nitric acid, and deionized water with a volume ratio of 1:2:7 for 2 min, and then repeatedly washed using deionized water. Subsequently, the treated titanium foil was put into a stainless-steel autoclave lined with polytetrafluoroethylene containing 9.08 g of KOH and 50 mL of deionized water. The hydrothermal reaction was performed at 220 °C for 24 h. After cooling to room temperature, the excess alkali solution and loose sample were washed repeatedly with deionized water, and then soaked in 30 mL of 0.6 M of hydrochloric acid for 30 min, and then the excess hydrochloric acid solution was washed again. The final titanium-supported K_x_Ti_y_O_2_ sample was obtained after a pyrolysis process in a muffle furnace at 400 °C for 1 h. The mass of the loaded active substance as the K_2_Ti_8_O_17_ electrode was approximately 1.0 mg cm^−2^.

### 2.3. Preparation of AC Electrode

The AC electrode was prepared by coating AC slurry on a 2.0 × 2.0 cm^2^ titanium mesh. The slurry was composed of AC, polytetrafluoroethylene (PTFE, 1 wt. %), and acetylene black (AB) with the mass ratio of AC:PTFE:AB = 8:1:1, uniformly dispersed in anhydrous ethanol. After the alcohol volatilized, the AC slurry was evenly coated on the titanium mesh, and the titanium mesh coated with AC was dried at 80 °C for 12 h to obtain the final AC cathode electrode. The mass of loaded active substance of the AC electrode was about 8 mg cm^−2^.

### 2.4. Physical Characterization of K_2_Ti_8_O_17_ Electrode

The phase structure of the K_2_Ti_8_O_17_ electrode was characterized by X-ray diffraction (XRD, Bruker AXS D8 (Akishima, Tokyo, Japan) diffractometer with Cu K_α_ radiation with λ of 0.15418 nm). To obtain a clear diffraction peak, it was necessary to scrape the sample off the Ti substrate and test the electron diffraction patterns of the powder sample. The morphology of the K_2_Ti_8_O_17_ electrode was characterized by field-emission scanning electron microscopy (FESEM, SUPRA55, Carl Zeiss Corp., Jena, Germany), transmission electron microscopy (TEM, HT7700, Hitachi Corp., Tokyo, Japan, 100 kV, 10 μA), and high-resolution TEM (HRTEM, FEI Titan 80~300 kV, FEI Corp., Hillsboro, OR, USA). The K_2_Ti_8_O_17_ electrode was cut into small pieces to stick onto the conductive tape for SEM measurement. A drop of slurry, uniformly suspended in ethanol, was dispersed onto the amorphous carbon film supported on a Cu grid for TEM and HRTEM analysis. Elemental analysis of the cycled K_2_Ti_8_O_17_ electrode was carried out on the ICP-OES system (730, Agilent, Palo Alto, CA, USA). The obtained sample (20 mg) was dissolved into 5 mL of concentrated nitric acid and then 25 mL of deionized water was added for the ICP-OES test. K-edge X-ray absorption near-edge spectroscopy (XANES) measurements (Beijing, China) of Ti in L-edge and O K-edges were performed at the 4B7B soft X-ray experiment station of the Beijing Electron Positron Collider (BEPC) (Beijing, China). Powder samples were mounted on electrically conducting carbon tape. 

### 2.5. Electrochemical Properties of the K_2_Ti_8_O_17_ and AC Electrodes

The electrochemical properties of the K_2_Ti_8_O_17_ and AC electrodes were evaluated by cyclic voltammetry (CV) and galvanostatic charge–discharge (GCD) based on the CHI 604E electrochemical station (CH Instruments Corp., Shanghai, China) and Land CT 2001A instruments (Land Instruments Corp., Wuhan, China), respectively. All the measurements were performed by the three-electrode system both in the 1 M AlCl_3_ solution and the mixed solution of 1 M of AlCl_3_ and NaAc. The saturated calomel electrode (SCE, all the voltage values in this paper are herein referred to as SCE) and the 1 × 1 cm^2^ Pt foil were used as the reference electrode and counter electrode, respectively. To further evaluate the practical application of the K_2_Ti_8_O_17_ electrode in the aluminum ion electrolyte, an AIB battery was assembled using AC as the cathode electrode pasted onto the titanium mesh (K_2_Ti_8_O_17_/Ti, 2 × 2 cm^2^) and K_2_Ti_8_O_17_ as the anode electrode (1 × 2 cm^2^) in the hybrid electrolyte of 1 M of AlCl_3_ and 3 M of NaAc (noted as AlCl_3_/NaAc). 

## 3. Results and Discussion

**Figure 1:**
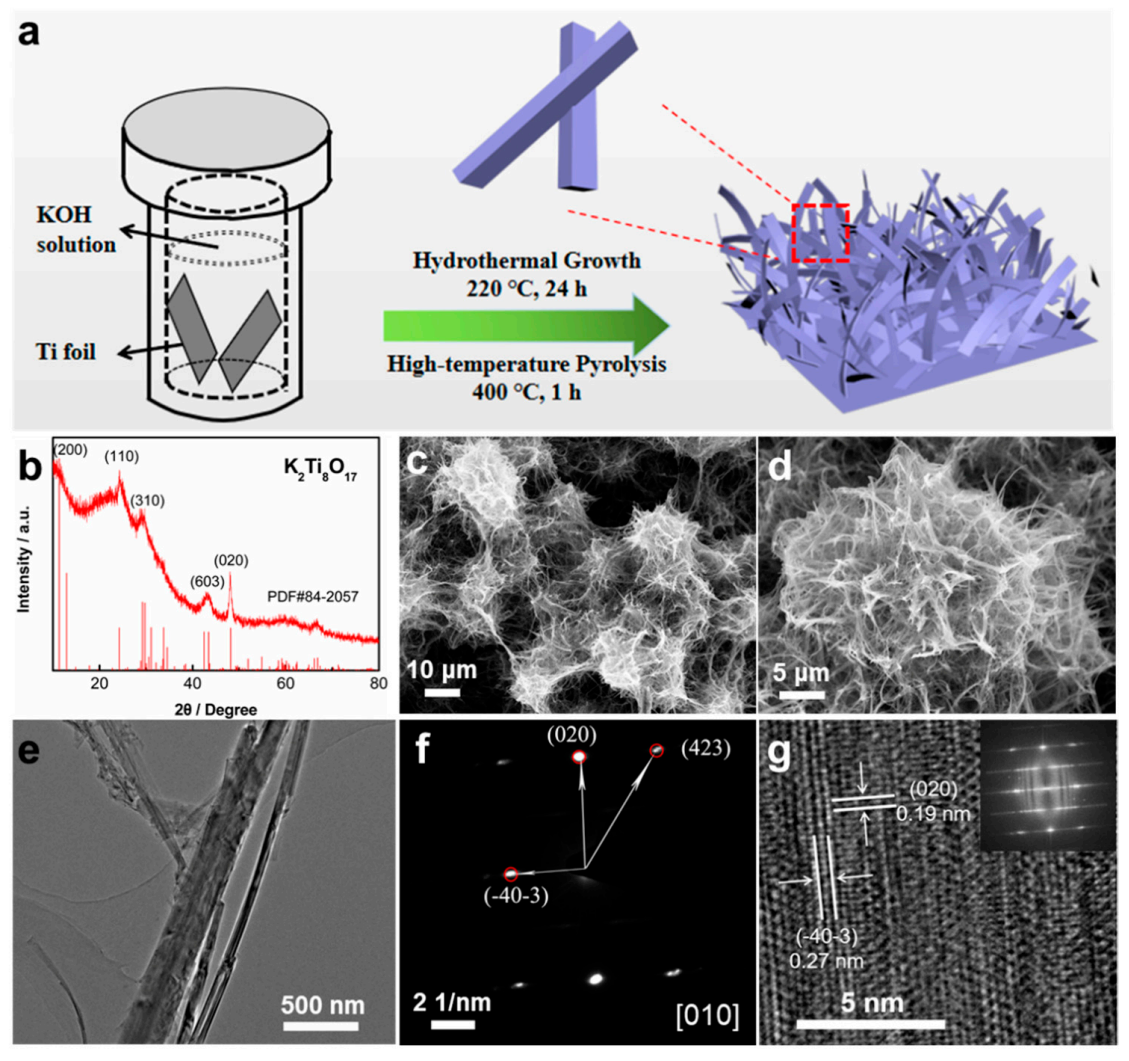
(**a**) Schematic of the preparation of K_2_Ti_8_O_17_ electrode via a hydrothermal method combined with a post-pyrolysis process. (**b**) XRD patterns of the K_2_Ti_8_O_17_ sample, (**c**) low- and (**d**) high-magnification SEM images of the TiO_2_ NWAs film. (**e**) TEM image of the K_2_Ti_8_O_17_ powder peeled from the titanium substrate. (**f**) Selected-area electron diffraction (SAED) pattern and (**g**) HRTEM image of the K_2_Ti_8_O_17_ sample.

As shown in the schematic (Figure 1a), the nanobelt K_2_Ti_8_O_17_ is formed on the surface of the Ti foil via the facile hydrothermal reaction combined with a post-pyrolysis process. Figure 1b displays the XRD patterns of the as-synthesized K_2_Ti_8_O_17_ nanobelts. All diffraction peaks are indexed as a layered K_2_Ti_8_O_17_ phase (JCPDF NO. 84-2057) with the space group of C2/m(12). The SEM image shows a network distribution of whiskers-like K_2_Ti_8_O_17_ (Figure 1c). The magnified image shows the nanoclusters aggregated with the intertwined nanobelts (Figure 1d). The formed pore structure inside the nanoclusters facilitates the electrolyte to reach the crystal structure. The TEM image in Figure 1e reveals an individual K_2_Ti_8_O_17_ nanobelt with a length of several microns. The SAED pattern and HRTEM image (Figure 1f,g) indicate a single crystal structure of the prepared K_2_Ti_8_O_17_ nanobelt. The growth direction of the nanobelt is along the [010] direction. The lattice plane distances of 0.19 nm and 0.27 nm correspond to (020) and ($\bar{4}0\bar{3}$) lattice planes of K_2_Ti_8_O_17_ (Figure 1g), respectively. Even though a good contrast can be observed in the SAED pattern (Figure 1f), the FFT (fast Fourier transform) pattern of the HRTEM image inserted in Figure 1g reveals an elongation of the diffraction spot that may indicate the existence of defects in the single crystal. 

**Figure 2:**
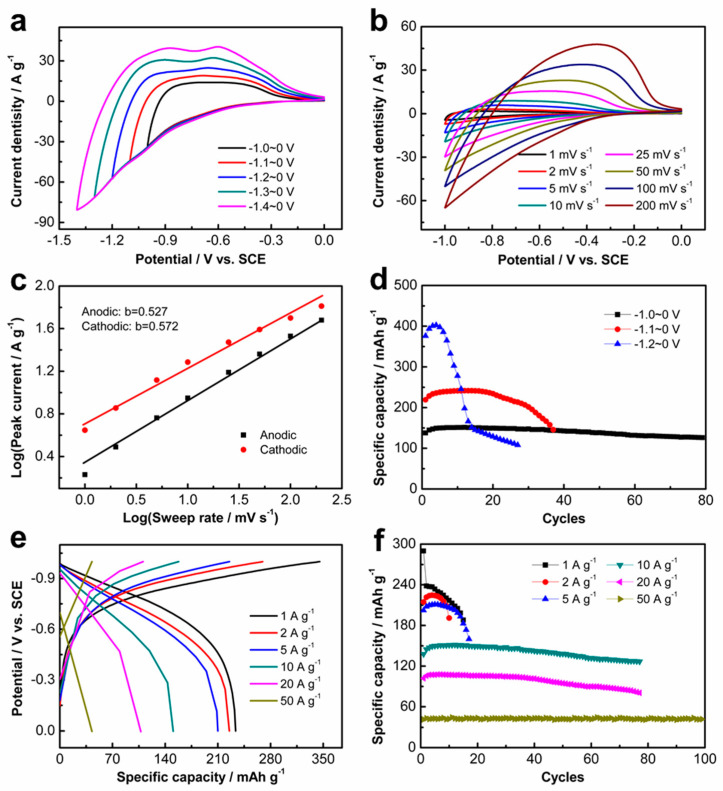
Electrochemical performance of K_2_Ti_8_O_17_ electrode in the AlCl_3_ aqueous solution: (**a**) CV curves at different potentials with sweep rate of 25 mV s^−1^. (**b**) CV curves with different scan rates at the potential range of −1~0 V_VS. SCE_. (**c**) Corresponding dependency of b-value for redox peak currents on the logarithm of the sweep rates. (**d**) Cycling performance in areal specific capacities by GCD within different potential ranges at current density of 10 A g^−1^. (**e**) GCD curves with different current densities in the potential range of −1~0 V_VS. SCE_. (**f**) Cycling performance at different current densities in the potential range of −1.0~0 V_VS. SCE_.

The electrochemical properties of the as-prepared K_2_Ti_8_O_17_ electrode were first investigated in 1 M of AlCl_3_ aqueous electrolyte via cyclic voltammetry (CV) and galvanostatic charge–discharge (GCD) curves at room temperature (25 °C). Figure 2a displays the CV curves at different potential ranges with a sweep rate of 25 mV s^−1^, showing that the K_2_Ti_8_O_17_ electrode can be operated at the negative potential range from 0.0 V to −1.4 V_VS. SCE_. An ultra-high overpotential for the hydrogen evolution reaction (HER) is observed after the Al^3+^ ions intercalation, indicating that the Al^3+^ ions intercalation is prior to the HER process. The ultra-high hydrogen evolution overpotential is ascribed to the low catalytic activity of both K_2_Ti_8_O_17_ nanobelts and Ti substrate for HER [22]. Al^3+^ ions with a smaller radius than that of Li^+^ ions facilitate the intercalation/deintercalation reactions for the charge–discharge process, inducing the high reversibility of the insertion/extraction of Al^3+^ ions into/from the anatase TiO_2_ electrode [15]. Similarly, it is possible that the Al^3+^ ions could insert/extract into/from the K_2_Ti_8_O_17_ electrode. Charge and discharge plateaus are observed in GCD curves at different potential ranges (Figure S1a), in good agreement with the CV curves. CV measurements were performed at different scan rates, as shown in Figure 2b. The peak currents of the K_2_Ti_8_O_17_ electrode gradually increase with the scan rates. Under different sweep rates, the CV curves of the layered K_2_Ti_8_O_17_ electrode exhibit similar quasi-symmetric shapes, illustrating the good reversibility of the Al^3+^ ions insertion/extraction. The electrochemical behavior of Al^3+^ ions in the K_2_Ti_8_O_17_ electrode was evaluated by analyzing the collected CV data at various sweep rates according to the following equation:

(1) $$i=av^{b}$$

where *i* is the measured current and *v* represents the sweep rate. Both *a* and *b* refer to the adjustable parameters. The *b*-values are determined from the slope by plotting log *i* vs. log *v*. Well-defined *b* values approaching 1 indicate capacitive characteristics, while *b* values close to 0.5 represent diffusion-controlled characteristics of the electrode [23]. Here, the *b* values were determined as 0.527 and 0.572 from the redox peaks (Figure 2c), indicating that the intercalation/deintercalation of Al^3+^ ions in the K_2_Ti_8_O_17_ electrode is a diffusion-controlled process. Figure 2d presents the cycling performance of the K_2_Ti_8_O_17_ electrode at different potential ranges with a current density of 10 A g^−1^. As shown, the specific capacity increases with the potential ranges, while the stability decreases. The similar shape of the charge and discharge curves also reveals the good reversibility of the K_2_Ti_8_O_17_ electrode (Figure 2e). The coulombic efficiencies (CEs) of the electrode at the current density of 10 A g^−1^ decrease with the cycling number (Figure S1b), implying the degradation of the reversibility with the increase in cycles. The cycling stability under various current densities at −1.0~0 V_vs. SCE_ is shown in Figure 2f. The K_2_Ti_8_O_17_ electrode exhibits a specific capacity of 224 mAh g^−1^ at the current density of 2 A g^−1^ with the expense of cycling stability. The specific capacity of 107 mAh g^−1^ is maintained at the current density of 20 A g^−1^. It is surprising that the specific capacity is still 43 mAh g^−1^ at the current density of 50 A g^−1^ with a superior cycling performance. The CEs of the K_2_Ti_8_O_17_ electrode reach up to approximately 100% at the high discharge current density of 50 A g^−1^ (Figure S1c). These results indicate the high-rate performance of the layered K_2_Ti_8_O_17_ electrode, which should be ascribed to the layered structure for the fast diffusion of Al^3+^ ions. Additionally, the morphology evolution process of the precursors of the K_2_Ti_8_O_17_ electrode with the hydrothermal reaction time was investigated via changing the hydrothermal time (0 h, 4 h, 8 h, 12 h, and 24 h). The SEM and XRD results are presented in Figure S2 in the supporting information. The compositional characterizations and electrochemical performance of the K_2_Ti_8_O_17_ sample undergoing a 24 h hydrothermal process without the pyrolysis step are shown in Figure S3 in the supporting information. Compared with the K_2_Ti_8_O_17_ sample before and after undergoing the pyrolysis step, the obviously enhanced electrochemical performance is achieved for the one undergoing pyrolysis. 

**Figure 3:**
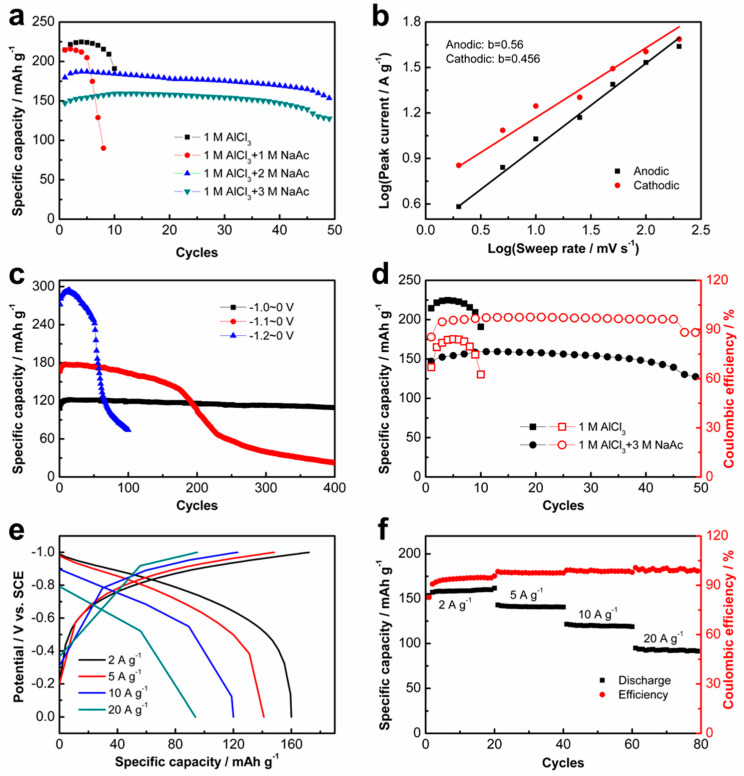
Electrochemical performance of K_2_Ti_8_O_17_ electrode in the AlCl_3_/NaAc aqueous electrolyte. (**a**) CV curves at different potential ranges with scan rate of 25 mV s^−^^1^. (**b**) Corresponding dependency of b-value for redox peak currents on the logarithm of the sweep rates calculated based on CV curves in Figure S4b. (**c**) GCD curves with different potential ranges at the current density of 10 A g^−1^. (**d**) Cycling performance in areal specific capacitances with current density of 2 A g^−1^. (**e**) GCD curves with different current densities in the potential range from −1 to 0 V_VS. SCE_. (**f**) Discharge capacitance and corresponding coulombic efficiencies at current densities ranging from 2 to 20 A g^−1^ in a potential range from −1 to 0 V_VS. SCE_.

To alleviate the effect of the acidic AlCl_3_ electrolyte on the electrode, various concentrations of the NaAc was added into the AlCl_3_ electrolyte as a buffer agent. The electrochemical performance of the K_2_Ti_8_O_17_ electrode in the hybrid AlCl_3_/NaAc aqueous electrolyte was evaluated by CV and GCD measurements (Figure 3 and Figure S4). The CV curves at different potential ranges show the symmetric shapes (Figure S4a), implying the good reversibility of the AIBs with the K_2_Ti_8_O_17_ anode in the hybrid AlCl_3_/NaAc aqueous electrolyte. Compared with the battery with pure AlCl_3_ electrolyte, the cycling stability of the battery under a current density of 2 A g^−1^ improves via the addition of NaAc with the concentrations of 2 M and 3 M (Figure 3a). The corresponding CEs based on the GCD tests also improve with the addition of NaAc solution (Figure S4b), although the discharge capacity of the battery slightly decreases (Figure S4c). This demonstrates that the NaAc additive is a benefit for the rechargeable intercalation/deintercalation of Al^3+^ ions. The b value plots for log i against log v of the K_2_Ti_8_O_17_ anode were calculated on the basis of the CV curves at different scan rates (Figure S4d) to obtain the b values via Equation 1. The calculated b values are 0.56 and 0.505 in the AlCl_3_/NaAc hybrid electrolyte (Figure 3b), suggesting a diffusion-controlled process in the K_2_Ti_8_O_17_ electrode [11,24]. The cycling performance of the charge–discharge curves of the K_2_Ti_8_O_17_ electrode in the aqueous AlCl_3_/NaAc electrolyte shows a similar trend to that in the AlCl_3_ electrolyte (Figure 3c and Figure S5a), exhibiting a decreasing trend with the potential ranges. In contrast, the cycling stability in the AlCl_3_/NaAc hybrid electrolyte is improved compared with that in the single AlCl_3_ electrolyte. To analyze the elemental composition of the cycled electrode, ICP-OES experiments for the cycled K_2_Ti_8_O_17_ electrodes in both charge and discharge states, as well the tested electrolyte, were performed. The cycled K_2_Ti_8_O_17_ electrode in the charge state was attained when it was charged to −1 V after charging/discharging for 10 cycles. The electrode in the discharge state was obtained when it was discharged to 0 V after charging/discharging for 10 cycles, and the tested electrolyte was collected simultaneously. All the samples were immersed into deionized water overnight and then repetitively washed before the ICP-OES test. From the experimental results, the molar ratio of Ti:K:Al:Na is 1:0.084:0.011:0.003 with the total cation charge of K, Al, and Na of around 0.96 in the charge state, while it is 1:0.087:0.016:0.002 with the total cation charge of K, Al, and Na of around 1.096 under the discharge state. The difference in K^+^ ions under charge and discharge conditions indicates the contributions from K^+^ insertion/deintercalation. Additionally, the difference of 0.136 in terms of the total cation charge of K, Al, and Na implies that the proton seems to also contribute to the charge/discharge process. Obviously, a decrease in the K^+^ ion is detected in the cycled electrode, while the Al^3+^ ion and a trace of the Na^+^ ion are detected. Therefore, we can conclude that the ionic substitutions of K^+^ ions by Al^3+^/Na^+^ ions occur in the K_2_Ti_8_O_17_ electrode during the charge/discharge process. Simultaneously, the K^+^ ions are detected in the cycled electrolyte, corresponding to the dissolution of K^+^ from the electrode to electrolyte during the intercalation of Al^3+^ and Na^+^ ions. The ICP-OES measurement for the cycled K_2_Ti_8_O_17_ electrode after washing shows the existence of Na^+^ and Al^3+^ ions. This indicates a possible mechanism of Na^+^ and Al^3+^ co-intercalation/deintercalation during the charge/discharge process. Although the specific capacitance of the K_2_Ti_8_O_17_ electrode in the aqueous AlCl_3_/NaAc electrolyte (159 mAh g^−1^) is lower than that in the AlCl_3_ electrolyte (234 mAh g^−1^), the cycling stability and CEs during the cycling are significantly improved (Figure 3d). Similar charge and discharge curve shapes reveal the excellent reversibility of the K_2_Ti_8_O_17_ electrode in the AlCl_3_/NaAc hybrid electrolyte (Figure 3e). The K_2_Ti_8_O_17_ electrode exhibits a superior rate capability and delivers good discharge capacities of 159, 141, 120, and 93 mAh g^−1^ with increasing current densities of 2, 5, 10, and 20 A g^−1^, respectively (Figure 3f). The corresponding CEs at the current densities of 2, 5, 10, and 20 A g^−1^ are approximately 90%, 93%, 94%, and 95%, respectively. These results demonstrate excellent rate-performance of the K_2_Ti_8_O_17_ electrode in the AlCl_3_/NaAc electrolyte. It is interesting that the cycling performance and CEs of the K_2_Ti_8_O_17_ electrode are improved with the increase in the charge–discharge current density (Figure S5b,c). The K_2_Ti_8_O_17_ electrode exhibited good cycling stability when the electrode performed in the potential range of −1.0~0 V at a current density of 10 A g^−1^ (Figure S5d), implying the promising practical application of the electrode at high current densities. Definitely, the electrolyte also has a disparate influence on different anodes [25,26].

**Figure 4:**
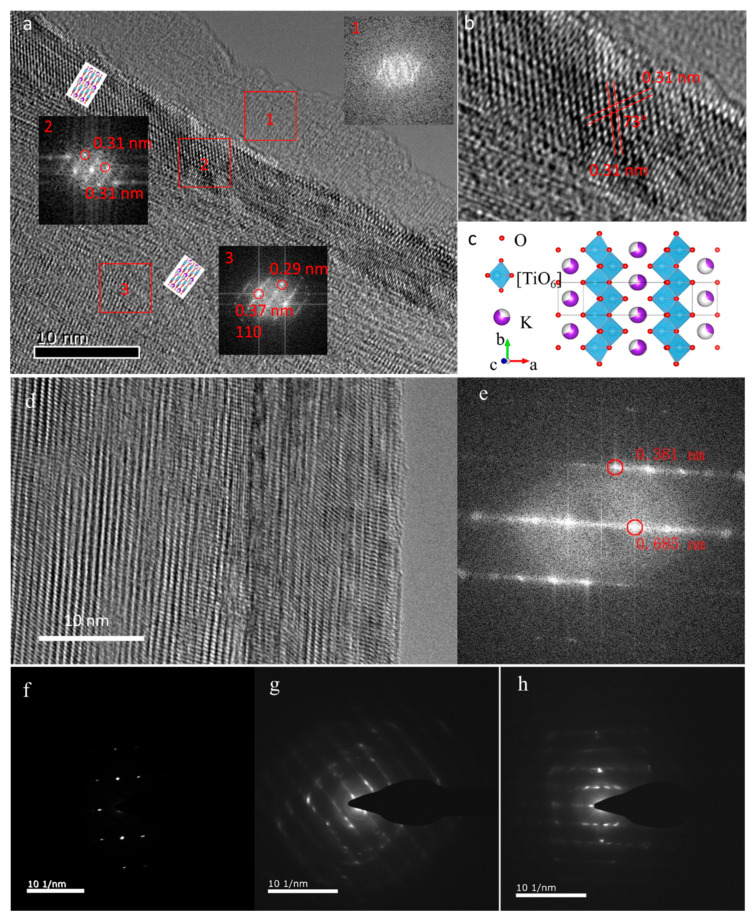
(**a**) HRTEM images of K_2_Ti_8_O_17_ electrode after charge–discharge cycling tests in aqueous AlCl_3_ electrolyte. (**b**) Magnified HRTEM image taken from red square box region 2 in (**a**). (**c**) Crystal structure of the layered K_2_Ti_8_O_17_. (**d**) HRTEM image and (**e**) corresponding FFT image of crystal structure of the layered K_2_Ti_8_O_17_ after cycling in the AlCl_3_/NaAc electrolyte for 100 cycles. SAED images of the layered K_2_Ti_8_O_17_ electrode, (**f**) pristine sample, (**g**) measured in the AlCl_3_ electrolyte, and (**h**) measured in the AlCl_3_/NaAc electrolyte.

To further probe the microstructure evolution of the K_2_Ti_8_O_17_ electrode during the charge–discharge cycling process in both the pure AlCl_3_ and the AlCl_3_/NaAc hybrid electrolytes, HRTEM measurements were performed and are shown in Figure 4. Three well-defined regions are presented in the K_2_Ti_8_O_17_ electrode after cycling in the aqueous AlCl_3_ electrolyte (Figure 4a). After electrochemical activation during the charge–discharge process, the phase transition occurs in the surface region due to the Na^+^/Al^3+^ intercalation/deintercalation in the K_2_Ti_8_O_17_ lattice, forming an amorphous phase (region 1 in Figure 4a). The corresponding fast Fourier transform (FFT) pattern confirms the amorphous structure (inset 1 of Figure 4a). Meanwhile, the well-defined crystalline lattice is still observed in the bulk of the K_2_Ti_8_O_17_ (region 3 in Figure 4a). The calculated lattice fringe distance of 0.37 nm via FFT corresponds to the (110) plane of the layered K_2_Ti_8_O_17_ phase. Between region 1 and 3, there is a crystallized layer that reveals a different FFT pattern from that of the bulk (region 2 in Figure 4a). The difference could be the orientation change of the K_2_Ti_8_O_17_ phase during the charge–discharge cycling process. The lattice plane distances of 0.31 nm, as well as the 73° cross angle of the lattice plane shown in Figure 4b, indicate that the lattice planes in region 2 are (310) and (−310) planes of the K_2_Ti_8_O_17_ phase. In the sample without the charge–discharge cycling process, no such layered structure is observed (see Figure 1f). The amorphization in region 1 and the reorientation in region 2 are ascribed to the irreversible phase transition of K_2_Ti_8_O_17_ during the charge–discharge process. The irreversible phase transition-induced amorphization of the surface layer of K_2_Ti_8_O_17_ primarily causes the capacity to fade. Analogously, the Al^3+^ ions intercalation and irreversible phase transition caused capacity degradation, which were also present in the transition-metal chalcogen for AIBs [12,27]. 

For comparison, the amorphous layer is not present in the K_2_Ti_8_O_17_ electrode after cycling in the hybrid AlCl_3_/NaAc electrolyte. Accordingly, the clear lattice fringe in the HRTEM image and the corresponding FFT plot (Figure 4g) indicate the relative high crystallinity in the sample, referring to the layered K_2_Ti_8_O_17_ phase. Selected-area electron diffraction (SAED) was employed to probe the phase structure of the K_2_Ti_8_O_17_ nanobelt before and after cycling. The three SAED images in Figure 4f–h show the pristine K_2_Ti_8_O_17_, as well as the K_2_Ti_8_O_17_ after cycling in the AlCl_3_ aqueous electrolyte and in the AlCl_3_/NaAc hybrid electrolyte, respectively. The pristine K_2_Ti_8_O_17_ nanobelt is a single crystal with high crystallinity (Figure 4f). After charge/discharge cycling, defects are introduced in the K_2_Ti_8_O_17_, causing local structure distortions. As shown in Figure 4g,h, the diffraction patterns become blurred after cycling in both AlCl_3_ and AlCl_3_/NaAc electrolytes, confirming the structure distortions. The distortion extent of the K_2_Ti_8_O_17_ electrode in the AlCl_3_/NaAc electrolyte is lower than that in the AlCl_3_ electrolyte. The trend of polycrystallization is also confirmed by the appearance of a diffraction ring. This could be attributed to the Na^+^/Al^3+^ intercalation into the layered K_2_Ti_8_O_17_ crystal structure during the charge–discharge process. In addition, the low-magnification SEM, energy-dispersive X-ray spectroscopy (EDS), and element mapping images of the pristine and reacted K_2_Ti_8_O_17_ electrodes provide information on the homogenous distribution of Al into the K_2_Ti_8_O_17_ phase (Figure S6). These results suggest that the Al^3+^ ions are successfully inserted into the layered K_2_Ti_8_O_17_ structure after the charge/discharge behaviors. However, no amorphous layer is found in the reacted K_2_Ti_8_O_17_ electrode in the hybrid AlCl_3_/NaAc electrolyte, implying the contributions of the additive NaAc on maintaining the crystallinity of the K_2_Ti_8_O_17_ electrode during the electrochemical process. Simultaneously, a significantly improved cycling performance of the K_2_Ti_8_O_17_ electrode in the hybrid AlCl_3_/NaAc electrolyte is obtained in comparison with that in the single AlCl_3_ electrolyte (Figure 3d). It should be ascribed to the ability of alkaline NaAc to change the AlCl_3_ aqueous electrolyte from neutral or acid conditions into alkaline conditions. As is known, the hydroxyl has a capacity to eliminate the formation of a Al_2_O_3_ passivation layer under alkaline conditions, while the formed Al_2_O_3_ passivation layer in neutral solutions hinders the electrode reactions, and a severe HER and self-discharge possibly occur under acidic conditions [12]. Therefore, we speculate that the NaAc additive eliminates the adverse effects of low pH values from the solo AlCl_3_ aqueous electrolyte, thus improving the electrochemical characteristics of the K_2_Ti_8_O_17_ electrode. Further evidence on the effect of the NaAc additive, however, is recommended in a study in the near future. As a comparison, the Na_2_Ti_9_O_19_ sample was synthesized using the same experimental method with K_2_Ti_8_O_17_. SEM, XRD, and electrochemical performance tests for the Na_2_Ti_9_O_19_ sample as an electrode in the AlCl_3_ electrolyte were conducted, as shown in Figure S7 in the supporting information. Compared to the K_2_Ti_8_O_17_ sample, the Na_2_Ti_9_O_19_ sample exhibits worse electrochemical performance from the CV curves and rate-capacity curves. 

**Figure 5:**
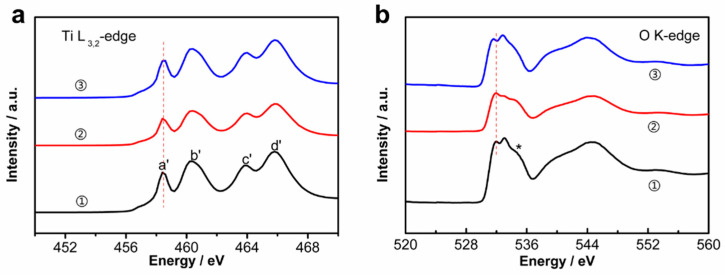
XANES spectra of (**a**) Ti L_3,2_-edge: the K_2_Ti_8_O_17_ electrode ① without testing, ② charged to 250 mAh, and ③ discharged after charging to 250 mAh; and (**b**) O K-edge of the pristine and undergoing various charge–discharge reactions of K_2_Ti_8_O_17_ electrode at the situations: ① without testing, ② charged to 250 mAh, and ③ discharged after charging to 250 mAh.

To gain insight into the reversibility of the electrode, an ex situ X-ray absorption near-edge structure (XANES) technique was employed here to identify the structural evolution of the K_2_Ti_8_O_17_ electrode materials during charge/discharge. Figure 5a displays the Ti L-edge XANES features undergoing the background subtraction and normalization procedures, for the pristine and tested K_2_Ti_8_O_17_ under various charge/discharge states. Four observed peaks located at 458.4 eV (a’), 460.7 eV (b’), 463.9 eV (c’), and 462.1 eV (d’) are ascribed to the excitation of Ti 2p^3/2^ (peaks t_2g_ (L3) and e_g_ (L3)) and Ti 2p^1/2^ (peaks t_2g_ (L2) and e_g_ (L2)) core levels into empty Ti 3d states, respectively [28,29,30]. As reported, the peak t_2g_ (L3) is related to the lower oxidation state of the cation, which has been detected on the TiO_2_ nanosheet with respect to the reduced Ti^4+^ surface specie [30]. The peak e_g_ (L3) is related to the higher oxidation state of the cation, corresponding to the Ti^4+^ species herein. The decreased intensity ratio of e_g_(L3)/t_2g_(L3) peaks (Table S1) after the discharge of the electrode indicate the partial reduction of Ti^4+^ during the discharge process. Moreover, differences between the pristine and the charged/discharged K_2_Ti_8_O_17_ electrodes are observed in the O K-edge XANES spectra (Figure 5b), reflecting the effects of Na^+^/Al^3+^ intercalation/deintercalation in the layered K_2_Ti_8_O_17_ electrode in the charge/discharge process. According to molecular orbital theory, the O K-edge features originate from a transition of the O 1s electron to the various partially occupied and unoccupied molecular orbitals of the oxides, considering the crystal-field splitting effects [31]. As shown in the O K-edge XANES spectrum of the pristine K_2_Ti_8_O_17_ sample, the major peaks located at about 532.6 eV and 534.1 eV are assigned to excitations varying from the O 1s core level to the Ti 3d-related conduction band, splitting into t_2g_ and e_g_ subbands [32]. This result confirms the partial reduction of Ti^4+^ during the discharge of the K_2_Ti_8_O_17_ electrode. 

**Figure 6:**
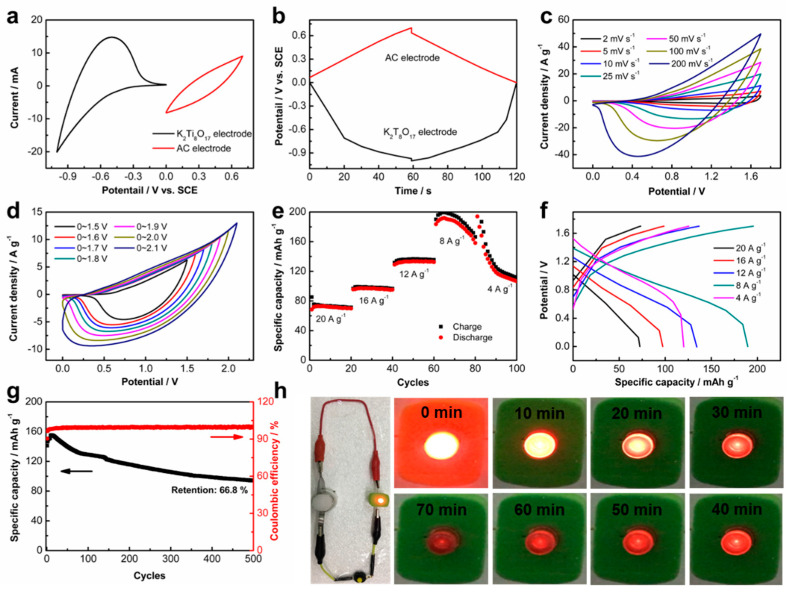
Electrochemical performance of AIB in the hybrid AlCl_3_/NaAc electrolyte: (**a**) CV curves of K_2_Ti_8_O_17_ electrode at the potential range of −1~0 V and AC electrode at the potential range of 0~0.7 V with a scan rate of 25 mV s^−1^; (**b**) GCD curves of the K_2_Ti_8_O_17_ electrode and AC-Ti electrode with the same current density of 20 A g^−1^ at the potential range of 0~1.7 V; (**c**) CV curves of the K_2_Ti_8_O_17_//AlCl_3_/NaAc//AC-Ti at different scan rates; (**d**) CV curves of the K_2_Ti_8_O_17_//AlCl_3_/NaAc//AC-Ti with a scan rate of 25 mV s^−1^ at different potential ranges; (**e**) specific capacitance of the K_2_Ti_8_O_17_//AC-Ti at different current densities; (**f**) GCD curves of the K_2_Ti_8_O_17_//AlCl_3_/NaAc//AC-Ti at different current densities; (**g**) cycling performance of the K_2_Ti_8_O_17_//AlCl_3_/NaAc//AC-Ti with the current density of 20 A g^−1^; (**h**) digital photographs of the K_2_Ti_8_O_17_//AlCl_3_/NaAc//AC-Ti-lighted LED indicator, and the lightness of the LED indicator lasting for different periods.

To further explore the potential practical application of the K_2_Ti_8_O_17_ electrode, a full cell of AIB was assembled using the carbon coated on the Ti mesh (AC-Ti), K_2_Ti_8_O_17_ electrode, and the aqueous AlCl_3_/NaAc hybrid solution as the cathode, anode, and electrolyte, respectively. The working potential range of the AC-Ti and K_2_Ti_8_O_17_ electrodes was measured by CV and GCD curves. The electrochemical properties of the AC-Ti electrode are given in Figure S8, demonstrating that AC-Ti is a suitable cathode for the aqueous AIB. Figure 6a presents the CV curves of both the cathode and anode. As shown, the AC-Ti electrode displays typical capacitance characteristics in the voltage range of 0~0.7 V_VS. SCE_, while the K_2_Ti_8_O_17_ electrode demonstrates the capacitance in the potential of −1~0 V_VS. SCE_ in the hybrid AlCl_3_/NaAc electrolyte. The AC-Ti cathode and K_2_Ti_8_O_17_ anode were also evaluated by GCD tests (Figure 6b). The potential ranges for the K_2_Ti_8_O_17_ and AC-Ti electrodes are consistent with the CV measurements. The maximal voltage of the as-assembled AIB device reaches up to 1.6 V, as shown in Figure 6c. The CV curves without any obvious shape distortions at various sweeping rates, even at a high sweeping rate of 200 mV s^−1^, indicate its superior rate capability. No gas evolution peaks are observed in the CV curves even at the potential range of 0~2.0 V_VS. SCE_ (Figure 6d). The specific capacities of the K_2_Ti_8_O_17_// AlCl_3_/NaAc//AC-Ti battery are 189.6, 134.3, 97.3, and 72.4 mAh g^−1^ at high scan rates of 8, 12, 16, and 20 A g^−1^, respectively (Figure 6d,f). Additionally, the AIB exhibits a capacity retention of approximately 66.8% after 500 cycles at the current density of 20 A g^−1^ (Figure 6g), demonstrating its superior cycling stability. Noticeably, a slightly larger discharge capacity is observed in the first 10 cycles and then decreases. Irreversible changes likely occurred in the electrode at the early stage [33,34]. Furthermore, the as-assembled AIB has the power to light-up a commercial light-emitting diode (LED) lasting more than one hour (Figure 6h), confirming its promising practical applications for energy storage. 

## 4. Conclusions

In conclusion, we developed a novel layered K_2_Ti_8_O_17_ anode material for an aqueous AIB via a facile hydrothermal process. The AIB was assembled with the as-prepared K_2_Ti_8_O_17_ electrode, AC-Ti, and AlCl_3_/NaAc hybrid aqueous solution as the anode, cathode, and electrolyte, respectively. The NaAc additive in the AlCl_3_ aqueous electrolyte played important roles in the enhanced electrochemical performance, improved rate performance, and optimized cycling stability. HRTEM and XANES techniques were conducted to gain deep insight into the Na^+^/Al^3+^ ion intercalation/deintercalation mechanism during charge and discharge processes. ICP-OES results indicate the multi-ions insertion/deintercalation contributions to the charge/discharge process in this AIB device. The designed aqueous AIB with the layered K_2_Ti_8_O_17_ anode and hybrid AlCl_3_/NaAc electrolyte exhibited a superior rate capability. Particularly, an excellent discharge capacity for this AIB was achieved up to 189.6 mAh g^−1^, even at a current density of 8 A g^−1^. This work therefore provides promising prospects for developing high-capacity and super rate-capability aqueous AIBs.

## Supplementary Materials

The following are available online at https://www.mdpi.com/article/10.3390/nano11092332/s1, Figure S1: The morphology evolution process of K_2_Ti_8_O_17_ electrode with the hydrothermal reaction times: (a) 0 h, (b) 4 h, (c) 8 h, (d) 12 h, and (e) 24 h, and (f) the XRD patterns of the K_2_Ti_8_O_17_ samples with different hydrothermal reaction times. Figure S2: (a) The SEM and EDS results of the K_2_Ti_8_O_17_ sample undergoing a 24 h hydrothermal process before the pyrolysis step. The electrochemical measurements of K_2_Ti_8_O_17_ electrode in AlCl_3_ electrolyte: (b) CV curves at different scan rates, (c) GCD curves at different current densities, and (d) rate performance. Figure S3: Electrochemical performance of K_2_Ti_8_O_17_ electrode in the 1 M AlCl_3_ aqueous solution. (a) GCD curves at different potential ranges with sweep rate of 10 mV s^−1^. (b) Coulombic efficiencies based on the GCD curves with a current density of 10 A g^−1^ at different potential ranges. (c) Discharge efficiencies based on the GCD curves at different current densities with a potential range of −1~0 V_vs. SCE_. Figure S4: Electrochemical performance of K_2_Ti_8_O_17_ electrode in the AlCl_3_/NaAc aqueous electrolyte. (a) CV curves at different potential ranges with a scan rate of 10 mV s^−1^. (b) CV curves with different scan rates at the potential range of −1~0 V_vs. SCE_; Coulombic efficiencies based on the GCD curves with current density of 10 A g^−1^ at different potential ranges. (c) Coulombic efficiencies based on the GCD curves at different current densities with a potential range of −1~0 V_vs. SCE_. Figure S5: (a) Charge–discharge curves of K_2_Ti_8_O_17_ electrode at different potential ranges at the current density of 10 A g^−1^. (b) Cycling performances and (c) CEs of the K_2_Ti_8_O_17_ electrode at different current densities. (d) Cycling performance of K_2_Ti_8_O_17_ electrode at the current density of 10 A g^−1^. Figure S6: (a) EDX energy spectrum and element distribution of the pristine K_2_Ti_8_O_17_. (b) EDX spectrum and element distribution diagram of electrode after 400 cycles of current density of 10 A g^−1^ in AlCl_3_/NaAc aqueous electrolyte. Figure S7: The physical characterizations and electrochemical performance of Na_2_Ti_9_O_19_ sample: (a) XRD pattern, (b) SEM image, CV curves at different scan rates, and (d) rate capacity and CEs. Figure S8: (a) CV curves of the AC coated on the Ti mesh (AC-Ti) electrode at different potential ranges with a scan rate of 25 mV s^−1^. (b) CV curves of AC-Ti electrode with different scan rates at the potential range of 0~0.6 V. (c) GCD curves of the AC-Ti electrode at different current densities. (d) Rate performance and (e) cycling stability of the AC-Ti electrode at different current densities at the potential range of 0~0.7 V. Table S1: The ratio of the peak intensities of the e_g_(L3)/t_2g_(L3) in K_2_Ti_8_O_17_ electrode.
