# Supplementary material for: Novel K2Ti8O17 Anode via Na+/Al3+ Co-Intercalation Mechanism for Rechargeable Aqueous Al-Ion Battery with Superior Rate Capability
Source: Nanomaterials (Basel). 2021 Sep 8;11(9):2332. doi: 10.3390/nano11092332 (PMC8464791; doi:10.3390/nano11092332)
Supplement: Supplementary file 1 [file nanomaterials-11-02332-s001.zip › nanomaterials-1324811-supplementary.pdf]

nanomaterials

MDPI

Article

Novel $K_2Ti_8O_{17}$ Anode via Na^+/Al^{3+} Co-Intercalation Mechanism for Rechargeable Aqueous Al-Ion Battery with Superior Rate Capability

Qiangqiang Feng [1,†], Yanyan Liu [1,*,†], Jitong Yan [1], Wei Feng [1], Shaozheng Ji [2,3] and Yongfu Tang [1,*]

1 Hebei Key Laboratory of Applied Chemistry, College of Environmental and Chemical Engineering, Yanshan University, Qinhuangdao 066004, China; f2394713046@163.com (Q.F.); yanjitong@outlook.com (J.Y.); fw980240907@163.com (W.F.)
2 Materials and Nano Physics, School of Engineering Sciences, KTH Royal Institute of Technology, SE-100 44 Stockholm, Sweden; jshaoz@kth.se
3 School of Physics, Ultrafast Electron Microscopy Laboratory, Nankai University, Tianjin 300071, China
* Correspondence: liuyy@ysu.edu.cn (Y.L.); tangyongfu@ysu.edu.cn (Y.T.)
† These authors make equal contributions.

1. Supplementary Figures

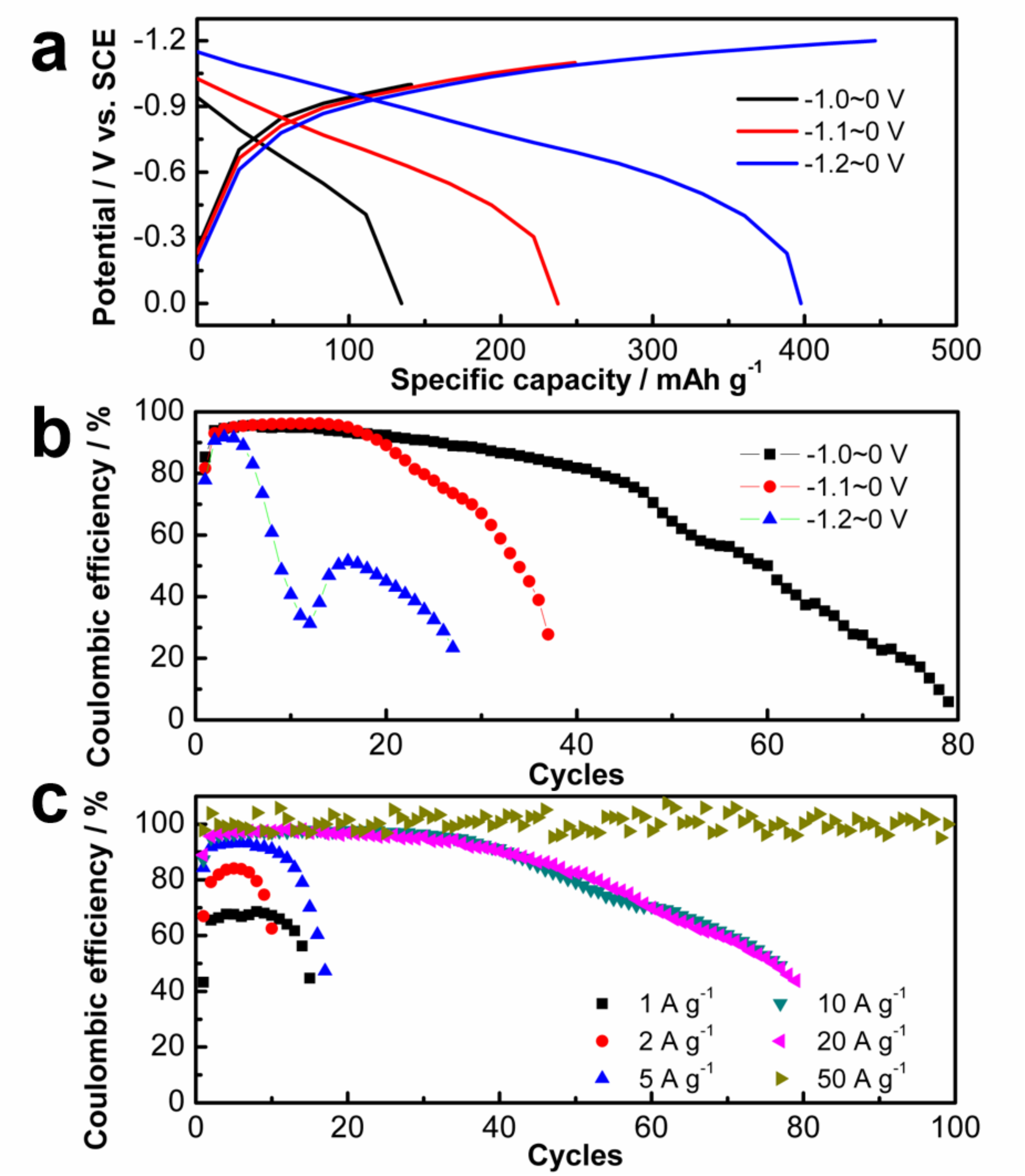

Figure S1. Electrochemical performance of $K_2Ti_8O_{17}$ electrode in the 1 M $AlCl_3$ aqueous solution. (**a**) GCD curves at different potential ranges with sweep rate of 10 mV s^{-1}. (**b**) Coulombic efficiencies based on the GCD curves with a current density of 10 A g^{-1} at different potential ranges. (**c**) Discharge efficiencies based on the GCD curves at different current densities with a potential range of −1~0 $V_{vs\ SCE}$.

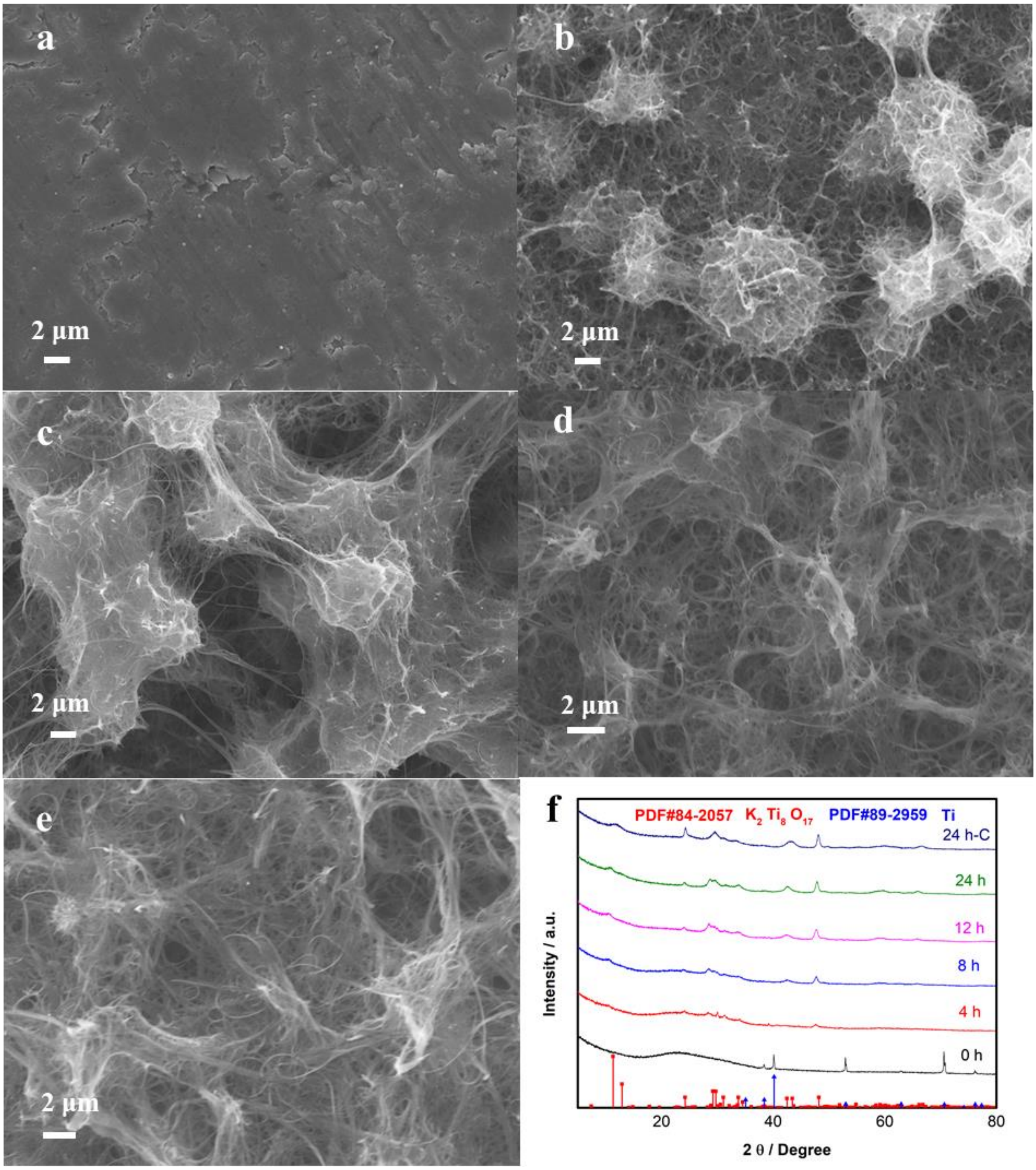

Figure S2. The morphology evolution process of $K_2Ti_8O_{17}$ electrode with the hydrothermal reaction times: (**a**) 0 h, (**b**) 4 h, (**c**) 8 h, (**d**) 12 h, (**e**) 24 h, and (**f**) the XRD patterns of the $K_2Ti_8O_{17}$ samples with different hydrothermal reaction times.

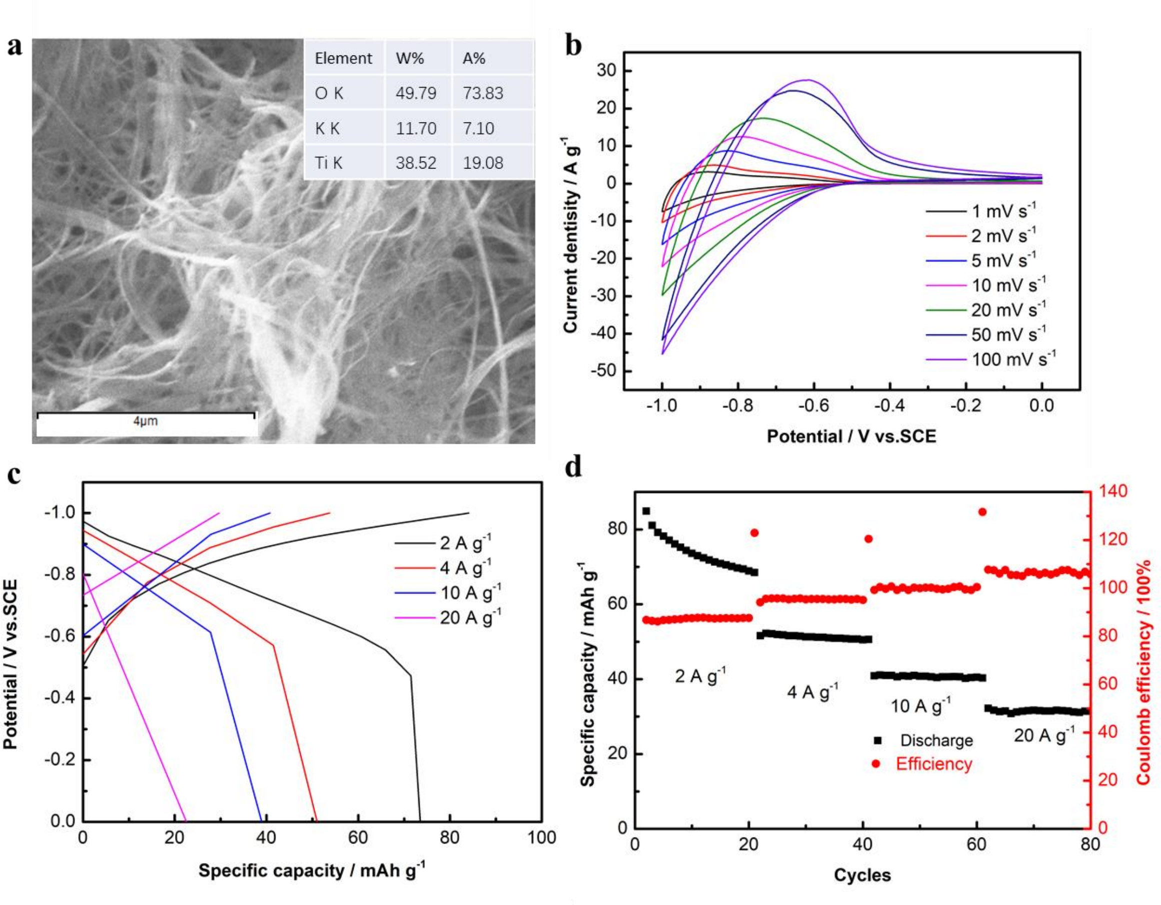

Figure S3. (**a**) The SEM and EDS results of the $K_2Ti_8O_{17}$ sample suffering a 24 h hydrothermal process before pyrolysis step. The electrochemical measurements of $K_2Ti_8O_{17}$ electrode in $AlCl_3$ electrolyte: (**b**) CV curves at different scan rates, (**c**) GCD curves at different current densities, (**d**) rate performance.

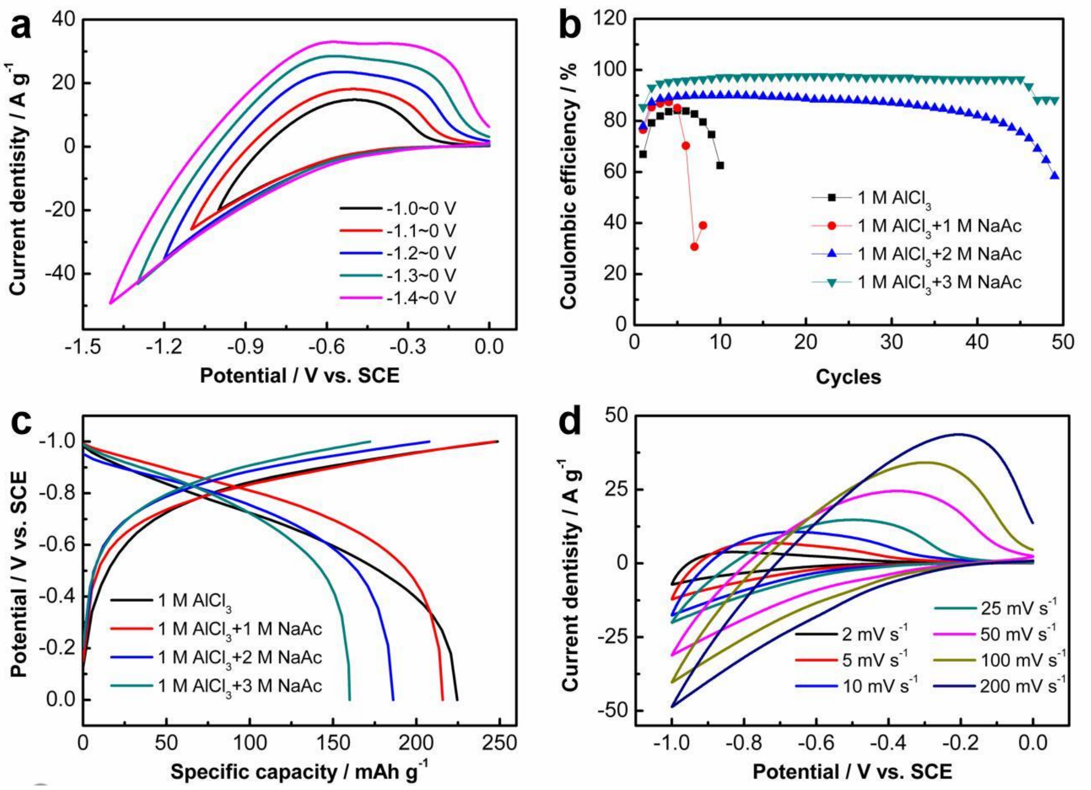

Figure S4. Electrochemical performance of $K_2Ti_8O_{17}$ electrode in the $AlCl_3$/NaAc aqueous electrolyte. (**a**) CV curves at different potential ranges with scan rate of 10 mV s^{-1}. (**b**) CV curves with different scan rates at the potential range of −1 ~ 0 $V_{vs\ SCE}$; Coulombic efficiencies based on the GCD curves with current density of 10 A g^{-1} at different potential ranges. (**c**) Coulombic efficiencies based on the GCD curves at different current densities with a potential range of -1~0 V $_{vs\ SCE}$. (d) the CV curves at various scan rates.

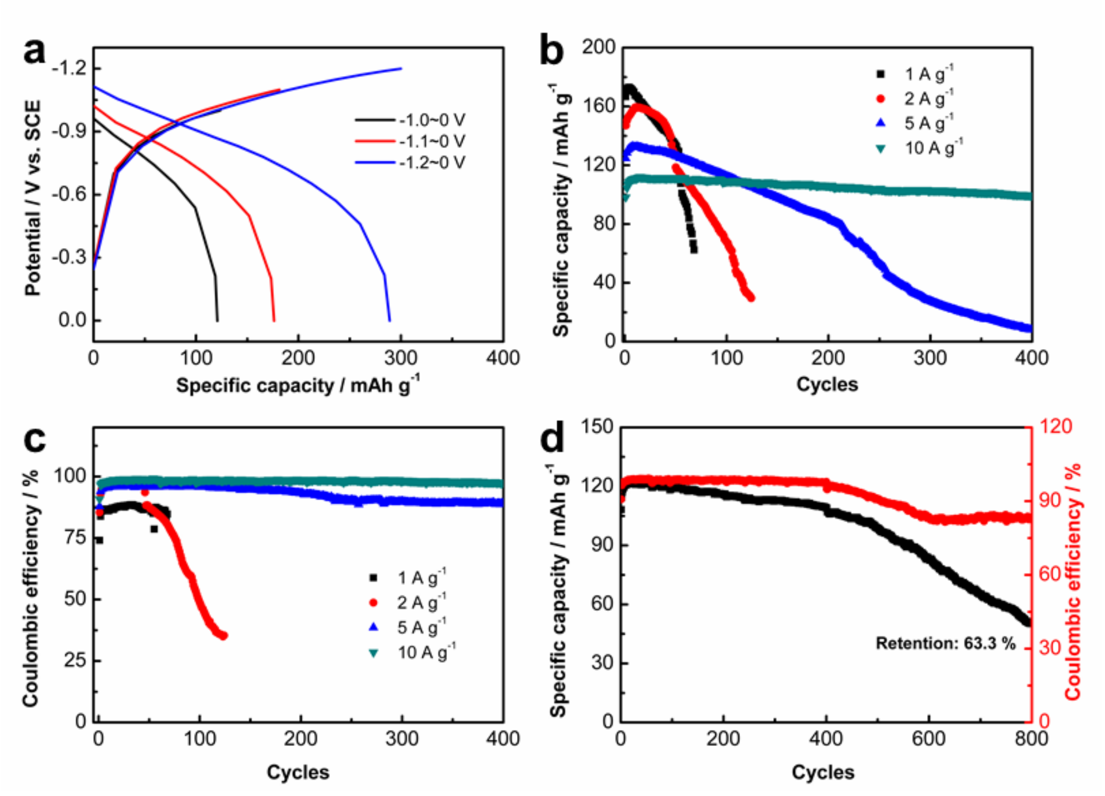

Figure S5. (**a**) Charge-discharge curves of $K_2Ti_8O_{17}$ electrode at different potential ranges at the current density of 10 A g^{-1}. (**b**) Cycling performances and (**c**) CEs of the $K_2Ti_8O_{17}$ electrode at different current densities. (**d**) Cycling performance of $K_2Ti_8O_{17}$ electrode at the current density of 10 A g^{-1}.

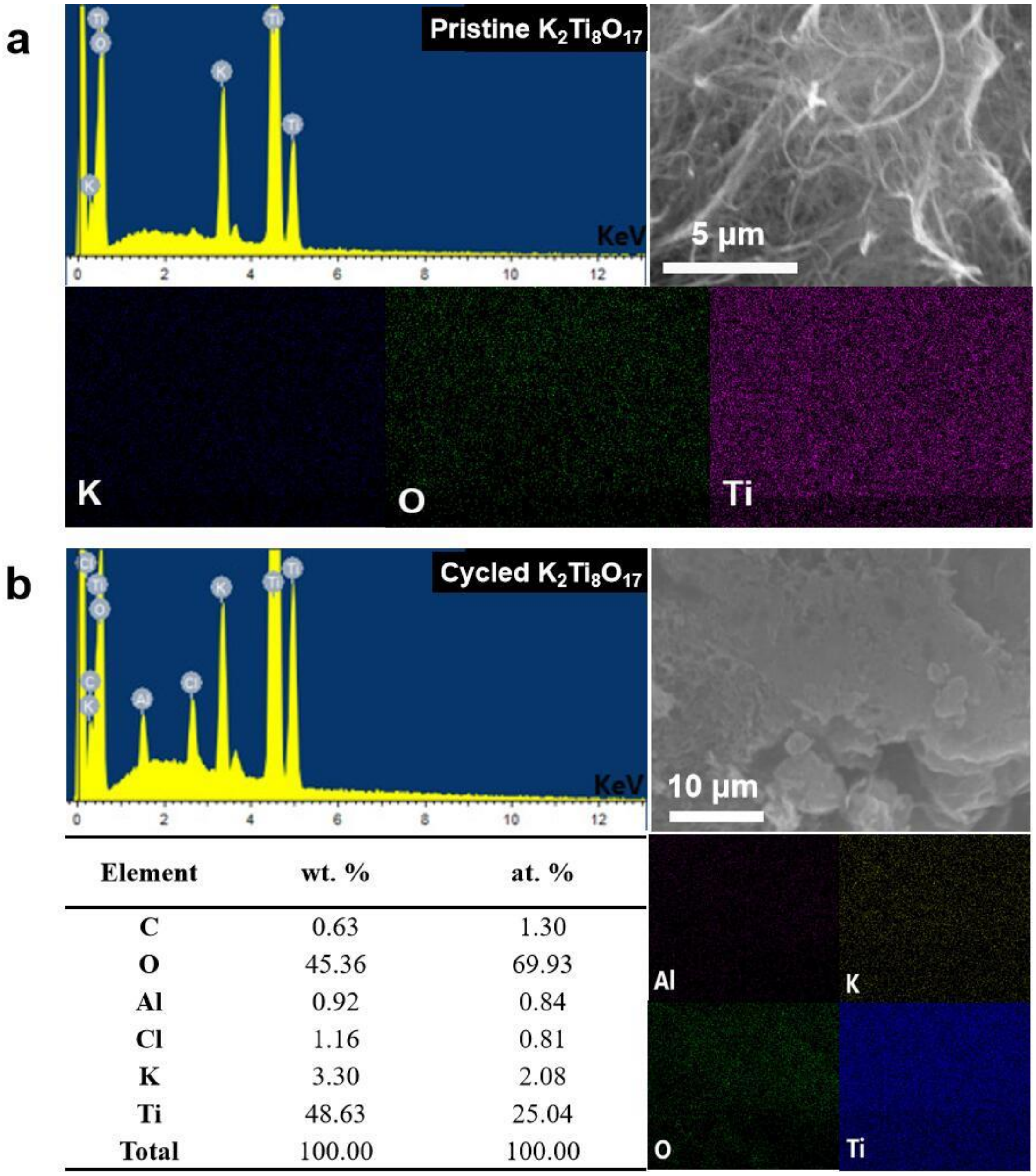

Element	wt. %	at. %
C	0.63	1.30
O	45.36	69.93
Al	0.92	0.84
Cl	1.16	0.81
K	3.30	2.08
Ti	48.63	25.04
Total	100.00	100.00

Figure S6. (**a**) EDX energy spectrum and element distribution of the pristine $K_2Ti_8O_{17}$. (**b**) EDX spectrum and element distribution diagram of electrode after 400 cycles of current density of 10 A g^{-1} in $AlCl_3$/NaAc aqueous electrolyte.

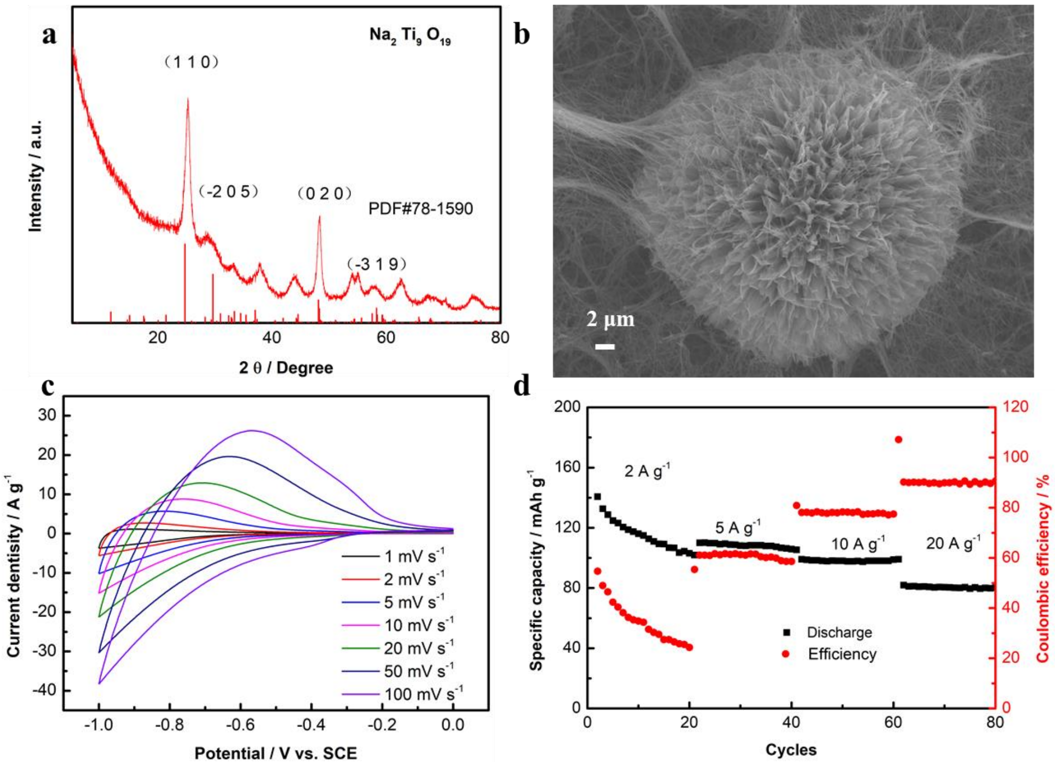

Figure S7. The physical characterizations and electrochemical performance of $Na_2Ti_9O_{19}$ sample: (**a**) XRD pattern, (**b**) SEM image, (**c**) CV curves at different scan rates, and (**d**) rate capacity and CEs.

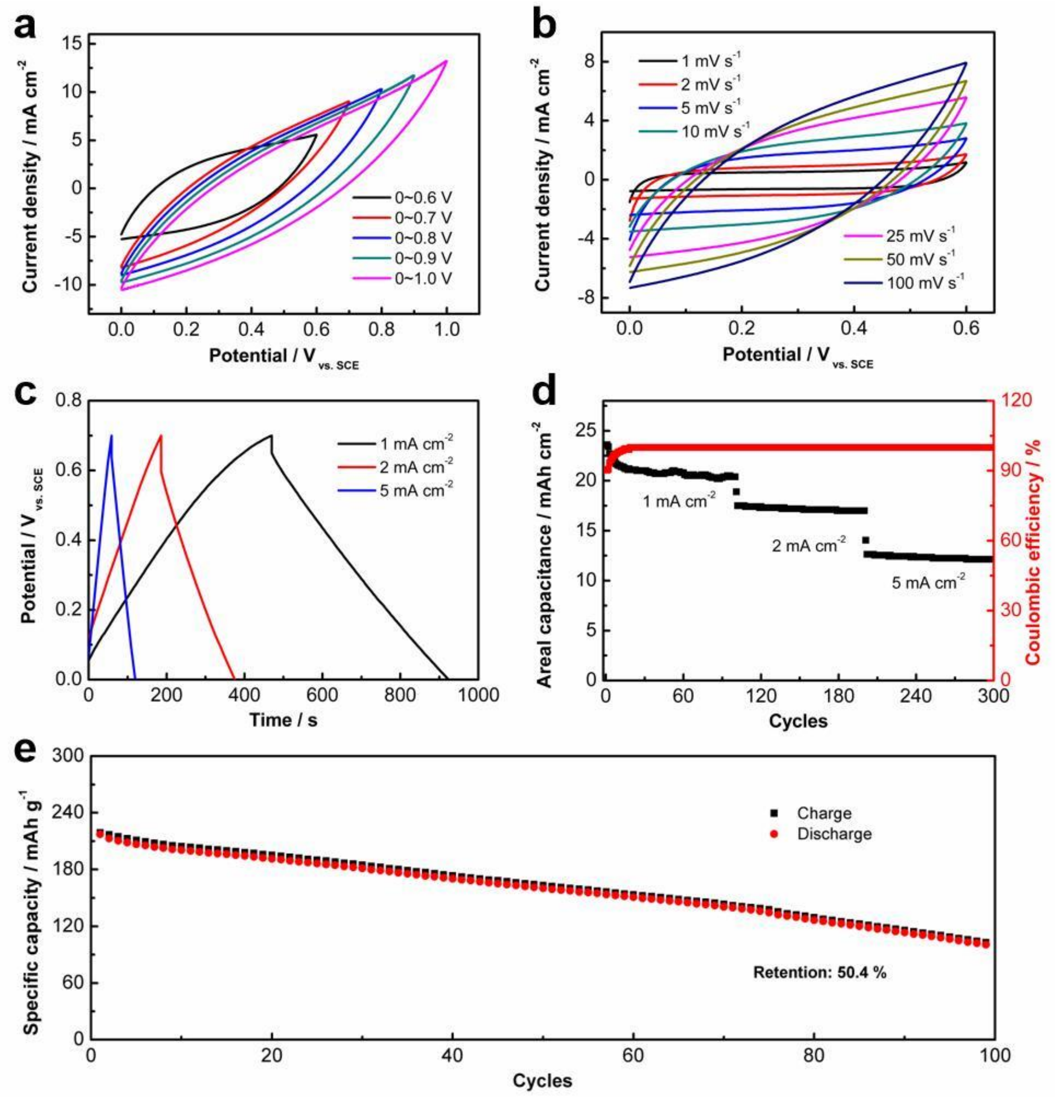

Figure S8. (**a**) CV curves of the AC coated on Ti mesh (AC-Ti) electrode at different potential ranges with Table 25. mV s^{-1}. (**b**) CV curves of AC-Ti electrode with different scan rates at the potential range of 0~0.6 V. (**c**) GCD curves of the AC-Ti electrode at different current densities. (**d**) Rate-performance and (**e**) cycling stability of the AC-Ti electrode at different current densities at the potential range of 0~0.7 V.

2. Supplementary Table

Table S1. The ratio of the peak intensities of the $e_g(L_3)/t_{2g}(L_3)$ in $K_2Ti_8O_{17}$ electrode.

Electrode	$K_2Ti_8O_{17}$	Discharge	Charge
$e_g(L_3)/t_{2g}(L_3)$	3.3590	3.3149	3.4906
